# Assessment of chronic ankle instability

**DOI:** 10.1186/1757-1146-5-S1-I2

**Published:** 2012-04-10

**Authors:** Kathryn M Refshauge, Claire E Hiller

**Affiliations:** 1 Faculty of Health Sciences, University of Sydney, Sydney, NSW 2141, Australia

## 

Chronic ankle instability is a complex problem which often follows ankle sprain. This workshop will discuss the latest models of chronic ankle instability and give an overview of the latest research in the area. Assessment tools available in the clinic as well as the laboratory setting will be demonstrated in the workshop with the opportunity for participants to practice. Assessments covered will include; balance, strength, joint laxity, motor control, and proprioception.
